# Sleep, Hormones, and Circadian Rhythms throughout the Menstrual Cycle in Healthy Women and Women with Premenstrual Dysphoric Disorder

**DOI:** 10.1155/2010/259345

**Published:** 2010-01-18

**Authors:** Ari Shechter, Diane B. Boivin

**Affiliations:** ^1^Centre for Study and Treatment of Circadian Rhythms, Department of Psychiatry, Douglas Mental Health University Institute, McGill University, Montreal, QC, Canada H4H 1R3; ^2^Integrated Program in Neuroscience, McGill University, Montreal, QC, Canada H3A 2B4

## Abstract

A relationship exists between the sleep-wake cycle and hormone secretion, which, in women, is further modulated by the menstrual cycle. This interaction can influence sleep across the menstrual cycle in healthy women and in women with premenstrual dysphoric disorder (PMDD), who experience specific alterations of circadian rhythms during their symptomatic luteal phase along with sleep disturbances during this time. This review will address the variation of sleep at different menstrual phases in healthy and PMDD women, as well as changes in circadian rhythms, with an emphasis on their relationship with female sex hormones. It will conclude with a brief discussion on nonpharmacological treatments of PMDD which use chronotherapeutic methods to realign circadian rhythms as a means of improving sleep and mood in these women.

## 1. Introduction

A variety of hormones, including melatonin, cortisol, thyroid stimulating hormone (TSH), and prolactin (PRL), vary across the 24-hour day and are highly regulated by the circadian and sleep-wake cycles. Evidence suggests that these hormones, as well as other physiological rhythms like body temperature, play a role in sleep organization and can also be affected by sleep itself (or lack thereof). These relationships can be further modulated by the menstrual cycle, since fluctuations in gonadotropic and sex steroid hormones occurring throughout the menstrual cycle can influence sleep, body temperature, and other hormones. 

Sleep disruptions are common in women, with reports of insomnia occurring 1.5–2 times more frequently than in men [1]. Indeed, sleep complaints commonly occur during the postovulatory luteal phase (LP) in healthy women [2]. These complaints reach a higher severity in women suffering from premenstrual dysphoric disorder (PMDD) [3], a DSM-IV classified menstrual cycle-related mood disorder. Since disturbed sleep and circadian rhythms have been correlated with increased incidence of obesity and diabetes [4], cardiovascular disease [5], and especially depression [6], and since depression already occurs with higher prevalence in women [7], it is necessary to understand how neuroendocrine changes across the menstrual cycle interact with circadian physiology and contribute to the greater susceptibility of sleep complaints in women. 

The aim of this paper is to review studies which investigated how the menstrual cycle, and its associated variation in sex steroid hormones, affects sleep and circadian rhythms in both healthy women and women with PMDD. Additionally, we will address the inconsistencies that often characterize these experimental results, highlighting methods which can minimize various confounders, and offer suggested areas of further research. Articles were included if they were written in English, conducted on human research participants, and concerned changes in sleep and/or circadian rhythms on at least two menstrual cycle phases in healthy and/or PMDD women. Though there were no date restrictions, menstrual cycle-related research articles included were published between 1984 and the present.

## 2. Hormones and the Sleep-Wake and Circadian Cycles

### 2.1. Circadian and Homeostatic Regulation of the Sleep-Wake Cycle

The sleep-wake cycle is regulated by an interaction between homeostatic (process S) and circadian (process C) processes [10]. Throughout the course of the waking day, the homeostatic drive for sleep pressure increases and dissipates rapidly during the subsequent sleep episode. This process has been linked to the restorative aspects of sleep and is quantifiable with the amount of slow wave sleep (SWS; stage 3 + 4 sleep based on standard polysomnographic sleep analyses [11]) or more accurately slow wave activity (SWA; power density within the 0.5–4.5 Hz frequency range based on spectral analysis of the EEG signal), which was demonstrated to increase as a function of the duration of prior awakening [12]. It was hypothesized that increasing levels of adenosine in the basal forebrain during waking contributes to the buildup of the homeostatic drive for sleep [9]. 

**Figure 1:**
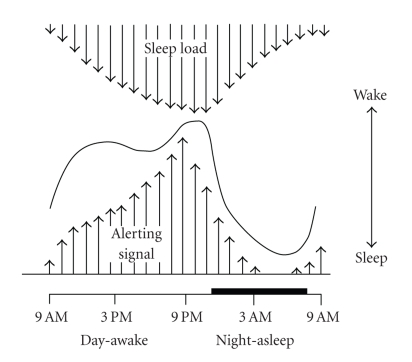
The interaction between circadian (C) and homeostatic (S) processes in an “opponent-process” results in an uninterrupted 8-hour nocturnal sleep episode and a wake period maintained throughout the 16-hour day. The homeostatic drive for sleep (illustrated as the “sleep load”) increases throughout the waking period and reaches a peak just before habitual bedtime. The circadian drive for alertness (illustrated as the “alerting signal”) reaches a peak at this time and is lowest near the end of the sleep episode. From [8].

At certain times of day, for example, just before habitual bedtime when the homeostatic drive for sleep is at its peak, and conversely at the end of the sleep episode when it is at its lowest, a strong circadian drive for wakefulness and sleepiness, respectively, counteracts process S. This interaction, referred to as the “opponent process,” results in uninterrupted 8-hour nocturnal sleep and 16-hour waking episodes each day (Figure 1) [8, 13]. Circadian rhythms (i.e., endogenously generated biological rhythms of about 24 hours) are observable in many aspects of human physiology and behavior, including neuroendocrine secretion [14], sleep propensity and architecture [10], and subjective and EEG-based estimates of alertness [15]. The suprachiasmatic nucleus (SCN) of the anterior hypothalamus is the master circadian pacemaker [16] and coordinates endogenous physiology with the external light-dark environment [17]. Sleep parameters including sleep onset latency (SOL), sleep efficiency (SE), rapid eye movement (REM) sleep, REM sleep onset latency (ROL), and spindle frequency activity (SFA; spectral power density within the 12–15 Hz range) show a strong circadian modulation [10].

**Figure 2:**
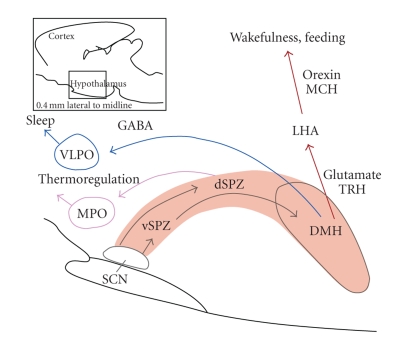
Pathways involved in the hypothalamic control of the circadian rhythms of sleep, wakefulness and body temperature. In the regulation of circadian sleep-wake patterns, outputs from the SCN relay at the vSPZ, and project to the DMH. The DMH then sends outputs to the VLPO (a sleep-activating center), and the LHA (where orexin neurons target downstream wake-promoting sites). The SCN regulates circadian body temperature rhythms through a relay at the dSPZ, which projects to the MPO. SCN, suprachiasmatic nucleus; vSPZ: ventral subparaventricular zone; dSPZ: dorsal subparaventricular zone; DMH: dorsomedial nucleus; VLPO: ventrolateral preoptic nucleus; LHA: lateral hypothalamic area; MPO: medial preoptic nucleus; MCH: melanin-concentrating hormone; TRH: thyrotropin-releasing hormone. Modified with permission from [9].

Signals originating in the SCN generate the circadian variation of sleep and wakefulness via major outputs to the ventral subparaventricular zone (vSPZ) and dorsomedial nucleus (DMH) (Figure 2) [9]. Some key arousal centers involved in this regulation are the histaminergic tuberomammillary nucleus (TMN), the noradrenergic locus coeruleus (LC), and the serotonergic dorsal and median raphe nucleus. The principle hypothalamic center for sleep initiation is the ventrolateral preoptic nucleus (VLPO). Activity in the VLPO is driven by the SCN via its projections to the vSPZ and DMH (Figure 2) [9]. Orexin neurons originating in the lateral hypothalamic area also receive projections from the SCN via the vSPZ and DMH, and promote wakefulness through their inputs to the TMN, LC, and raphe nucleus [9]. The sleep-wake system is presumed to be dependent on the mutually inhibitory interaction between these key arousal and sleep centers [9]. According to this “flip-flop” model, sleep occurs when the VLPO dominates, whereas waking occurs when it is inhibited by histaminergic, noradrenergic and serotonergic inputs [9].

### 2.2. Circadian and Sleep-Wake Dependent Variation of Hormones

A variety of hormones cycle with a 24-hour rhythmicity, though some are more regulated by the endogenously generated circadian system, whereas others are more sensitive to the timing of sleep per se [14]. 

Melatonin and cortisol are two hormones which vary with a strong circadian component, and are therefore reliable markers of circadian phase, or the timing of the central circadian oscillator [18]. The two have different times of peak amounts, with high melatonin levels throughout the biological night, during which cortisol levels are minimal. When cortisol peaks in the early morning, melatonin secretion is already declining to reach almost undetectable levels during the day [18]. Both hormones are sensitive to environmental factors like retinal light exposure (which suppresses melatonin secretion) and stress (which stimulates cortisol release). Thus to most accurately assess their circadian expression, it is advised to study them under constant conditions, which will reduce the occurrence of confounding “masking effects” on their secretion [19]. 

Other hormones such as TSH and PRL cycle with a 24-hour rhythmicity but are also sensitive to sleep-wake state. Under normally entrained conditions, TSH levels begin rising before the nocturnal sleep episode, and progressively decline throughout the sleep period [18]. Sleep has an inhibitory effect on TSH secretion [20]; therefore when sleep is prevented, TSH levels remain high throughout the nighttime hours. In comparison, PRL is stimulated by sleep [20], with peak amounts detectable during the sleep episode, and a minor, but significant endogenously generated circadian variation when sleep is eliminated [18].

### 2.3. Relationship between Melatonin, Body Temperature, and the Sleep-Wake System

**Figure 3:**
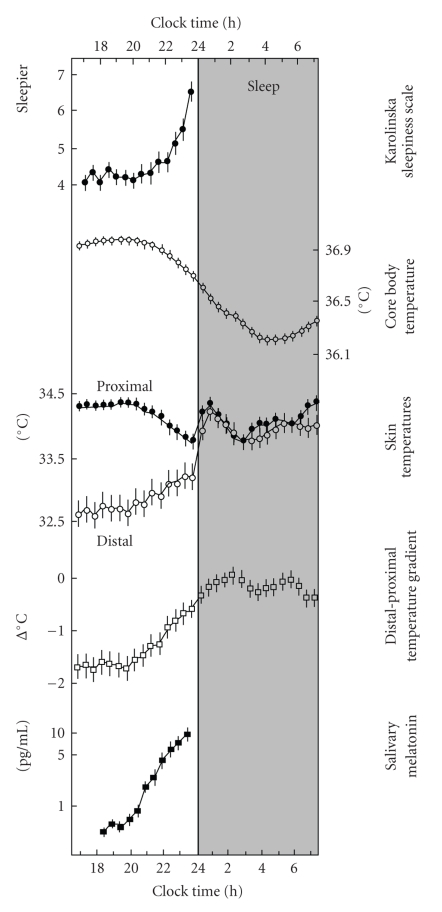
The relationship between melatonin secretion, body temperature and sleepiness. The onset of melatonin secretion during the early night causes an increase in heat loss at the extremities (i.e., rising distal skin temperature, and distal-proximal temperature gradient) and a drop in core body temperature, followed by an increase in sleepiness. From [21].

Melatonin levels vary concomitantly with body temperature and sleep propensity across the 24-hour day [21]. Specifically, under entrained conditions, the late evening rise in circulating melatonin levels triggers a thermoregulatory cascade, which, via an increase in the blood flow through distal skin regions and a subsequent decrease in core body temperature (CBT), favors sleep initiation (Figure 3) [21]. 

Core and distal body temperature levels show robust circadian rhythms, which are controlled by the SCN through projections to the dorsal subparaventricular zone (dSPZ) and ultimately the medial preoptic region (Figure 2) [9]. Constant routine experiments have illustrated this circadian variation for CBT, which reaches a peak in the late evening (21:00–22:00) and a trough during the latter part of the night (05:00–06:00) [24]. Distal skin temperature showed an inverse time course, that is advanced by 25–100 minutes with respect to the CBT curve [24]. Sleep is typically initiated on the declining limb of the CBT curve [25], and statistical regression analyses revealed that the distal-proximal temperature gradient (a measure of heat loss at the extremities) is the best predictor of a rapid SOL [26]. Exogenous melatonin administered during the day (when endogenous levels are low) reduces CBT and increases skin temperature, with concomitant increases in sleepiness [27]. These results indicate that melatonin may achieve its soporific effects through a thermoregulatory pathway. In addition to increasing sleepiness and sleep propensity, exogenous melatonin can affect sleep architecture [28–30], regardless of its effect on body temperature [31, 32]. These functional relationships and the localization of melatonin receptors throughout the brain and periphery [33] suggest that melatonin can affect the sleep-wake and circadian systems.

## 3. Normal Menstrual Cycle

### 3.1. Hormonal Regulation of the Menstrual Cycle

**Figure 4:**
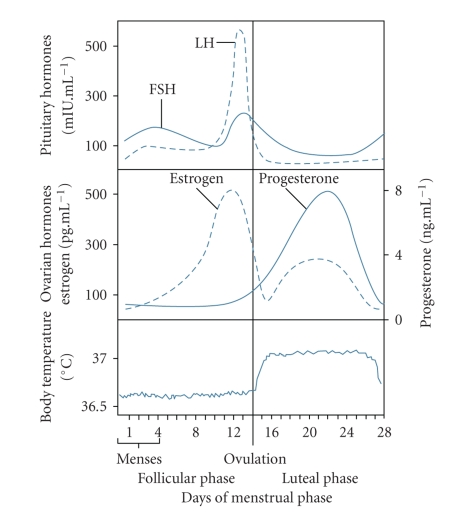
The variation of gonadotropic and sex steroid hormones, and the subsequent changes in daily body temperature across the full menstrual cycle. During the pre-ovulatory FP, estrogen levels are high. During the post-ovulatory LP, increasing levels of circulating progesterone are observed, along with increased daily body temperature. FSH, follicle stimulating hormone; LH, luteinizing hormone; FP, follicular phase; LP, luteal phase. From [22], as adapted from [23].

The menstrual cycle in healthy, ovulating females is regulated as well as defined by changes in the gonadotropic hormones, follicle-stimulating hormone (FSH) and luteinizing hormone (LH), and the sex steroid hormones, estrogen and progesterone (Figure 4). At the start of the cycle, during the pre-ovulatory follicular phase (FP), FSH stimulates ovarian follicles to grow and develop at which point circulating estrogen levels begin to rise and remain high throughout the FP. This culminates in ovulation at mid-cycle, when LH levels surge and stimulate the release of an oocyte. The subsequent secretion of sex hormones by the newly formed corpus luteum characterizes this post-ovulatory LP, when progesterone is the dominant hormone. If the egg is not fertilized, sex hormone levels drop at the end of the LP and trigger the shedding of the uteral lining (menstruation) [34].

### 3.2. Body Temperature Changes Associated with the Menstrual Cycle

Hormone changes across the menstrual cycle result in altered body temperature. Most notably, during the LP compared to the FP, there is an increase of ~0.3–0.4°C in CBT levels (Figure 4) [35, 36] as well as a significant reduction in the amplitude of the circadian variation of CBT [35–38], owing mainly to a blunted nocturnal decline of CBT. Skin temperature and vascular blood flow, which are important thermoregulatory responses, are affected by the menstrual cycle. Increased threshold for sweating [39, 40] and for vasodilation [39–41] as well as decreased thermal conductance and skin blood flow [42] is observed during the LP compared to the FP. 

This upward shift in the thermoregulatory set-point is most likely due to progesterone, which possesses thermogenic properties [36, 43], and was shown to increase the firing rate of cold-sensitive (i.e., body warming) neurons in the preoptic anterior hypothalamus (POAH) [44].

## 4. Sleep across the Menstrual Cycle in Healthy Women

### 4.1. Standard Polysomnographic Sleep

A relatively limited number of studies have addressed sleep-wake patterns across the menstrual cycle in healthy women. These have indicated that while sleep homeostasis [49, 51, 55] and quality [43, 49, 52] remain stable at different menstrual phases, there are observable changes in sleep architecture [49, 51–53] (summarized in Table 1). Interestingly, women often report subjective complaints of disturbed sleep during the late-LP and premenstrual days, though polysomnography- (PSG-) based estimates indicating disrupted sleep during this time are less frequent [22]. Since most studies compared sleep at only two menstrual phases (e.g., mid-FP versus mid- or late-LP), inconsistencies still remain regarding the variation of SWS [45, 47, 52, 53] and REM sleep [43, 47, 54, 55] across the menstrual cycle. 

In the first systematic study of sleep EEG across the menstrual cycle in healthy women, nocturnal sleep was recorded in the laboratory every other night throughout a full cycle [49]. This study showed no menstrual cycle-related change in SE (%), SOL (min), SWS (%) and wake after sleep onset (WASO; min) [49]. Non-REM (NREM) sleep and stage 2 sleep (%) significantly increased in the LP, while REM sleep (% of the NREM-REM sleep cycle) significantly decreased in the LP [49]. In a later study focusing on sleep-disordered breathing and the menstrual cycle, Driver et al. compared sleep at one visit during the FP and the LP [54]. They reported a significant increase in stage 2 sleep (%) during the LP, no change in SWS, and failed to replicate the significant decrease in REM sleep (%) generally reported during this phase [54]. 

A variety of studies compared sleep at either two or three phases of the menstrual cycle (mid-FP versus mid-LP [43, 53]; mid-FP versus late-LP [52, 55]; mid-FP versus mid-LP versus menses [51]). Across three phases, REM sleep (min) was significantly reduced during the mid-LP compared to the mid-FP, latency to stage 3 sleep was significantly reduced during the mid-LP compared to menses, and there were no significant changes observed for stage 2 sleep (min) or SWS [51]. Comparing the sleep of healthy women at the mid-FP and mid-LP, one study found no significant differences between any sleep parameter (including SE, SOL, REM sleep and SWS) [43], whereas another report detailed significantly decreased REM sleep (%) and significantly increased SWS (%) at the mid-LP compared to the mid-FP [53]. Focusing on healthy women and women with premenstrual syndrome (PMS) at the mid-FP and late-LP, Baker et al. reported that healthy women had significantly increased WASO (min) and microarousals per hour during the late-LP compared to the mid-FP, with no other significant changes observed between menstrual phases [55]. The results from eighteen healthy controls studied by Parry et al. at the mid-FP and late-LP as part of a larger study of sleep in PMDD found increases in ROL (min) and stage 1 sleep (%), and decreased REM sleep (min) and stage 3 (min and %) during the late-LP compared to the mid-FP [52]. 

Two investigations studied PSG sleep across four phases of the menstrual cycle [45, 47]. The first, which included eight healthy participants at the early-FP, the late-FP, the early-LP and the late-LP, found significant menstrual phase variations for stage 3 sleep (min), with a trough at the late-LP, and intermittent awakenings, with a peak at the late-LP [45]. The second, which included recordings of seven healthy females at menses, the late-FP, the early-LP and the late-LP, only found a significant variation for SWS (min), which, like the aforementioned study [45], was lowest during the LP compared to the late-FP and menses [47].

Interested in studying the effects of the menstrual cycle on the circadian variation of sleep propensity, Shibui et al. applied an ultra-rapid sleep-wake cycle procedure to eight healthy females at the FP and LP [37]. Sleep propensity (defined in the study as the sum of the duration of stages 2, 3, 4 and REM sleep occurring at each 10-minute nap trial) varied significantly across the circadian day, but did not differ between menstrual phases. Their main finding was that from 09:00 to 16:30, the number of naps containing SWS was increased during the LP compared to the FP [37]. It should be noted, however, that their participants were sleep-deprived for 24 hours preceding the start of the ultra-rapid sleep-wake cycle, creating a situation that could have increased the homeostatic pressure for SWS propensity, thus potentially confounding these results.

### 4.2. Quantitative Sleep EEG

The effects of menstrual phase on quantitative sleep EEG have been investigated by a few groups, yet results indicate a very consistent pattern of findings, making the prominent increase in SFA during the LP the most characteristic menstrual cycle associated sleep change [48, 49, 55] (Table 1). The sleep of five healthy young women was recorded by Ishizuka et al. at least three nights per week across a complete menstrual cycle [48]. Defining a sleep spindle as activity within the 11.11–16.13 Hz frequency range, the authors described a biphasic variation in the frequency of spindles, with lowest values observed during the FP (18 days before menstruation, near the mid-FP) and highest values during the late-LP [48]. Similarly, in the aforementioned study by Driver et al., which tracked sleep changes throughout an entire menstrual cycle in nine healthy women, SFA (here defined as mean power density within the 12.25–15.00 Hz frequency range) was lowest during the FP and reached peak values during the LP [49]. Maximum menstrual phase variation was observed within the 14.25–15.00 Hz band, and SWA (mean power density within the 0.75–4.50 Hz frequency range), a marker of sleep homeostasis, was unchanged across the menstrual cycle [49]. Finally, in the recent study by Baker et al., healthy women showed significantly increased SFA (12–15 Hz) during the late-LP compared to the mid-FP, with the most prominent peak again occurring in the 14.25–15.00 Hz bin specifically [55].

### 4.3. Summary and Future Steps

The most common sleep findings across the menstrual cycle include decreases in REM sleep, increases in stage 2 sleep and SFA, and no changes in sleep propensity and quality (SOL and SE, resp.) during the LP compared to the FP. Most studies agree with the absence of changes in homeostatic sleep mechanisms (i.e., SWS and SWA) at different menstrual cycle phases, although some inconsistencies remain (Table 1). Methodological differences between the various studies might contribute to these discrepancies. For example, menstrual phase delineation and the number of sleep recordings across the cycle is often different between studies, and menstrual phase status is not uniformly confirmed with hormonal assays. Stabilization of sleep-wake patterns before lab entry is not always done, even though it is recommended to ensure a proper alignment of sleep and circadian rhythms.

The changing sex hormone profile across the menstrual cycle may play a role in producing these LP-specific sleep alterations. Specifically, progesterone, as well as its neuroactive metabolites, can affect sleep architecture, as was illustrated by the findings that exogenous progesterone [69] or megestrol acetate, a progesterone-receptor agonist [70], reduced REM sleep in male participants. Likewise, exogenous progesterone in rats reduced REM sleep while lengthening ROL [71]. Furthermore, progesterone likely affects the sleep system through another indirect means, namely by increasing body temperature during the LP. Sleep architecture, like the timing of sleep propensity, is under a circadian regulation, with highest REM sleep occurring at times corresponding with the nadir of body temperature [72]. The finding of reduced REM sleep during the LP, when nocturnal body temperature is significantly elevated compared to the FP, is therefore interesting.

The LP-associated increase in SFA is most likely a result of the neuroactive metabolites of progesterone acting as agonistic modulators of central nervous system GABA_A_-receptors in a benzodiazepine-like manner [49, 71]. Indeed, progesterone administration enhanced spindle activity in the rat [71] and in male participants (particularly those who experienced an early allopregnanolone peak in response to exogenous progesterone treatment) during the first two hours of sleep [69]. Like REM sleep, the temporal pattern of SFA displays a robust circadian rhythm, with the peak of low-frequency SFA (12.25–13.00 Hz range) occurring during periods of high endogenous melatonin concentration, whereas high-frequency SFA (14.25–15.50 Hz range) is minimal during these times and the greatest during periods of low circulating melatonin [73]. 

The functional significance of increased SFA during the LP in women is still unknown. Since sleep spindles are thought to have a sleep-protecting effect via their blockage of information processing to the cortex [74], increased SFA may be the mechanism through which sleep quality is maintained at a good level despite the changing physiological and hormonal profile associated with different menstrual cycle phases.

## 5. Circadian Rhythms across the Menstrual Cycle in Healthy Women

It has been proposed that the menstrual cycle could form a backdrop on which daily circadian rhythms are expressed [22], and as such, circadian physiology can be altered as a function of the changing hormone profile associated with different menstrual phases (see Table 2 for a summary). The most apparent of these alterations is CBT (see above); yet other biological and hormonal rhythms, including melatonin, cortisol, TSH, and PRL may also be affected. It was proposed that one implication of the altered circadian rhythms observed during the menstrual cycle is the production of a stable intrauterine environment [35]. Specifically, the authors point to the reduced efficacy of melatonin function during the LP, which results in a blunted nocturnal decline of CBT and reduced circadian CBT amplitude, as a stabilizing factor which would encourage proper implantation and development of a fertilized egg [35]. However, these effects may also contribute to the increased incidence of subjective sleep complaints during the LP. 

### 5.1. Cortisol, TSH, and PRL across the Menstrual Cycle

A small number of studies looked at rhythms of cortisol, TSH, and PRL (Table 2). The circadian variation of cortisol in healthy women was found to be phase-delayed by ~1 hour [63], phase-advanced by ~1 hour [68] or decreased in amplitude [37] during the LP compared to the FP. PRL showed either a trend for increased amplitude during the LP compared to the FP [64] or no change across the menstrual cycle [60]. Sampling throughout an ultra-rapid sleep-wake cycle, the TSH rhythm was found to be decreased in amplitude and delayed by ~80 minutes in the LP compared to the FP [37]. Since limited number and inconsistencies once again characterize these data, it is important to replicate these studies using highly controlled experimental conditions and adequate sample sizes.

### 5.2. Melatonin across the Menstrual Cycle

Melatonin is known to play a role in reproductive physiology (see [75] for a review). Studying menstrual-related changes in melatonin secretion has been a topic of interest, though findings remain equivocal (Table 2). An early study sampling plasma melatonin every four hours during the FP and LP reported a significant increase in the total amount of secretion in 24 hours during the LP compared to the FP [58]. This result was supported by the finding that nocturnal urinary immunoreactive melatonin concentration (sampled nightly over an entire menstrual cycle) was significantly increased during the LP compared to the FP [59]. However, in a well-controlled study sampling every hour during the FP and LP under constant conditions, the 24-hour area under the curve (AUC) for plasma melatonin was significantly decreased during the LP, though other timing measures were unaffected [37]. On the other hand, in an important study outlining the role of melatonin on body temperature changes during the LP, Cagnacci et al. found that while AUC was unchanged between menstrual phases, there was a significant delay of ~110 minutes in the onset of nocturnal melatonin during the LP [35]. Most other studies have found no change in the patterns of melatonin secretion (including onset, offset, duration, midpoint, and AUC) across the menstrual cycle in healthy women [47, 60–62, 65, 67]. Furthermore, strengths of these studies were that they actually sampled melatonin across the menstrual cycle (i.e., at four menstrual phase [47, 61, 62, 65] as opposed to only two), or under constant conditions [67].

### 5.3. The Interaction between Sex Hormones and Melatonin

Evidence indicates that the pineal melatonin system and the reproductive system interact, as was illustrated by a variation in the number of cerebral and caudal arterial melatonin binding sites in the rat throughout the estrous cycle [76]. An interaction between the melatonin system and sex hormones may have an influence on sleep and body temperature rhythms across the menstrual cycle. Further support for such an interaction comes from the colocalization of melatonin receptors with estrogen and progesterone receptors throughout the brain and periphery. Specifically, considering areas involved with the reproductive cycle, melatonin binding sites were found at human [77] and rat [78, 79] granulosa cells, and melatonin was found in human ovarian follicular fluid [80]. Furthermore, various sources indicate that receptors for melatonin, progesterone, and estrogen can all be found at the SCN [81, 82], POAH [82, 83], and pineal gland [84, 85]. 

Evidence of a functional interaction between melatonin and sex hormones was presented by Cagnacci et al. in the aforementioned study, who illustrated that women experience a progesterone-dependent resistance to the hypothermic effects of melatonin during the LP [35]. While this appears to support a functional antagonism between melatonin and progesterone, there is also evidence for a positive relationship between the two. Exogenous synthetic progestins (in the form of oral contraceptives) have a tendency to increase melatonin secretion [58, 59, 67], and melatonin treatment can enhance human chorionic gonadotropin-stimulated progesterone production from human granulosa cells [86]. Conversely, estrogen appears to negatively influence melatonin. For example, a low-estrogen environment was associated with increased melatonin levels in menopausal women, which was suppressed after exogenous estrogen administration, and oopherectomy in premenopausal women results in a significant increase in melatonin secretion [87]. Estrogen treatment also reduced melatonin binding in the rat ovary [78] and reduced melatonin synthesis in rat pinealocytes [88].

### 5.4. Summary and Future Steps

Most groups have found no change in the circadian hormone profiles of melatonin, cortisol, TSH, and PRL, though phase-delays were observed for melatonin, cortisol, and TSH during the LP compared to the FP (Table 2). Interestingly, when TSH and PRL were found to change during the LP compared to the FP, the directions of these changes (i.e., decreased TSH amplitude and increased PRL amplitude) are the opposite of what occurs after a partial nocturnal sleep deprivation [89], though PSG-based estimates of sleep indicate total sleep time and SE are unchanged at different menstrual phases (see Table 1). 

Most studies which sampled hormones at different menstrual phases did not do so under controlled conditions, which are advised to limit the confounding effects of environmental factors (notably ambient light exposure, posture changes, and the sleep-wake cycle), something which likely contributes to these discrepancies [90]. Again, differences in the methods of dividing the menstrual cycle as well as sampling frequency (both across 24 hours and the menstrual cycle) are likely to contribute to inconsistencies in the literature. More studies need to be conducted before definitive conclusions can be made regarding the circadian variation of different hormone secretions across the menstrual cycle.

## 6. Premenstrual Dysphoric Disorder

### 6.1. Definition and Symptoms of PMDD

PMDD is a mood disorder affecting 3%–8% of North American women [91]. As is implied by its name, the occurrence of PMDD is defined by its timing within the context of the menstrual cycle. Symptoms typically begin during the late-LP and remit after menses, with a complete absence of symptoms during the FP. The DSM-IV lists a number of core symptoms for PMDD, including depressed mood, anxiety/tension, affective lability, anger/irritability, and decreased interest, each of which must reach sufficient severity to disrupt social, academic, or professional functioning [92]. Among these mood specific symptoms, sleep disturbances (including hypersomnia or insomnia, which is reported in as much as 70% of PMDD women [92]) are often present during the symptomatic LP. Since PMDD women may suffer from altered hormone secretion and/or function (see below), endocrinological factors and their influence on the sleep-wake system are important to consider when discussing this patient population.

### 6.2. Proposed Causes of PMDD

While the exact causes of PMDD are still unknown, a variety of hypotheses have been proposed which implicate endocrine or other neurotransmitter systems. An altered sex hormone profile in PMDD has been reported, with lower progesterone levels found in patients compared to controls [93, 94] as well as decreased levels of the anxiolytic progesterone metabolite allopregnanolone during the LP in patients [94, 95]. Progesterone produces its anxiolytic/hypnotic effects via allopregnanolone's binding to GABA_A_-receptors [96, 97], and some have found lower plasma GABA concentrations [98] and a decreased GABA_A_-receptor sensitivity [99] during the LP in PMDD patients compared to controls. Results of prior drug trials have found the most effective treatment of PMDD to date to be selective serotonin reuptake inhibitors (SSRIs) and they have become the most common clinical treatment for the disorder [100]. Experimental evidence implicating the serotonergic system includes findings of reduced plasma- [101] and whole-blood [102] serotonin levels in patients compared to controls. This raises the question of whether low serotonin levels could alter the production of melatonin by the pineal gland, since serotonin is a precursor for melatonin synthesis. Interestingly, PMDD patients experience alterations in the timing and amount of nocturnal melatonin secretion (see Section 8.2 below [62, 65]), though it is unclear whether this is a cause or a characteristic of the disorder.

## 7. Sleep across the Menstrual Cycle in PMDD Women

### 7.1. Standard Polysomnographic Sleep and Qualitative Sleep EEG

Although disrupted sleep is a characteristic symptom of PMDD, results of sleep studies in these women have been limited and inconsistent (Table 1). A preliminary study comparing six healthy controls and three patients with PMS (defined as a set of emotional, physical, and behavioral symptoms that occur with similar timing, but less severity, as PMDD) failed to detect significant differences in any sleep parameter [50]. A larger study with 23 PMDD patients and 18 controls also showed no intergroup differences, though significant menstrual phase effects were noted. In both groups, ROL (min) was increased, while REM sleep (min) and stage 3 (min and %) were decreased in the LP compared to the FP [52]. In a comparison of “premenstrually symptomatic” women (defined with an increase of at least 30% in the Profile of Mood States questionnaire during the LP) with controls, women experiencing negative mood symptoms during the LP showed decreased SWS (%) at both menstrual phases as well as decreased latency to stage 1 sleep and a trend for increased stage 2 sleep (%) in the LP [46]. Another study revealed that, compared to controls, PMS patients had more stage 2 sleep (%) and less REM sleep (%), and within these patients, stage 3 sleep (min; peaks near the late-FP/early-LP) and intermittent awakenings (peaks near the late-LP) varied significantly across the menstrual cycle [45]. More recently, a study including healthy women and those with PMS found decreased SWS (%) and REM sleep (%) as well as increased stage 2 (%) during the LP in both groups [56]. 

To date, one study [55] investigated quantitative sleep EEG in addition to standard PSG sleep in women with PMS/PMDD. Results from this comparative study showed that women with severe PMS and healthy controls both experienced similar increases in WASO (min) and microarousals per hour during the late-LP compared to the FP. Compared to controls, PMS/PMDD women showed increased ROL (min) in both menstrual phases. Similar to what has been shown for healthy controls, PMS/PMDD women demonstrated a menstrual variation for SFA (12–15 Hz), with marked increases during the late-LP. Interestingly, compared to controls, these women showed a trend for increased EEG activity in the 12-13 Hz range [55].

### 7.2. Summary and Future Steps

Within-patients studies of sleep across the menstrual cycle in PMS/PMDD patients revealed reduced REM sleep during the LP compared to the FP (Table 1). A significant menstrual cycle variation of stage 3 sleep was observed, and two other studies found decreased SWS or stage 3 sleep during the LP (Table 1). 

PMS/PMDD women were found to have increased WASO (min) and microarousals per hour during the LP compared to the FP, indicating more disturbed sleep during this symptomatic phase, but in that study, results were not different than healthy controls [55]. Similar to controls, PMS/PMDD women experienced a significant increase in SFA during the LP compared to the FP, though, here, a trend for increased activity in the 12-13 Hz range was observed for patients compared to controls (Table 1). Other comparisons of PMS/PMDD women and healthy control women showed patients to have increased stage 2 sleep, decreased REM sleep, or decreased SWS regardless of menstrual phase (Table 2). It remains unclear what could be causing PMDD-specific sleep changes, and further studies should address the relationships between sleep and parameters which are known to be altered in the PMDD patients, like CBT, melatonin concentration, and circadian phase. 

Increased SFA during the LP in PMDD women may serve a sleep-protective role that is similar to what is proposed for healthy women. The further increase in spindle activity within the 12–13 Hz range in PMS/PMDD, beyond what is observed in controls, may illustrate a strengthening of this effect, which is especially relevant since PMDD patients are most at risk for sleep disruptions during the LP. PMDD women also experience altered REM sleep, which is a hallmark of affective disorder [103]. Interestingly, a sleep restriction study in PMDD patients [52] (see Section 9.2) demonstrated a significant correlation between increasing REM sleep and improved mood, which implies that the reduced REM sleep sometimes observed in PMDD patients may contribute to symptom development. 

Important methodological issues should be addressed in these studies as well, including (in addition to those mentioned previously) the high degree of patient heterogeneity and diagnostic criteria used in these investigations. Only one study [52] to date has addressed sleep in a singular group of women whose diagnosis reached the DSM-IV standards to be defined as PMDD.

## 8. Circadian Rhythms across the Menstrual Cycle in PMDD Women

### 8.1. Body Temperature across the Menstrual Cycle in PMDD Women

Evidence suggests that PMDD patients can experience altered biological rhythms of body temperature and hormone secretion that could contribute to symptom development and/or exacerbation. An early study showed that, compared to healthy controls, PMDD women had significantly elevated nocturnal CBT and a reduced CBT amplitude during the LP [104]. Although a later study [105] failed to replicate these results, the authors described a decreased amplitude during the LP within PMDD patients, as well as a trend for increased nocturnal CBT in PMDD women compared to controls during the LP. Finally, a nonsignificant trend for a phase-advanced temperature minimum in PMDD patients compared to controls was observed across the entire menstrual cycle [45]. Differences in experimental techniques and data collection methods are likely contributors to inconsistencies in the aforementioned studies. For example, none of these controlled for the confounding effects of ambient light exposure, posture, and sleep or by utilizing a constant routine protocol to “unmask” the endogenous rhythm of CBT. Furthermore, patient diagnostic criteria, sample size, and the frequency of temperature recordings throughout the menstrual cycle all varied between the studies. Future research should consider these methodological issues.

### 8.2. Hormones across the Menstrual Cycle in PMDD Women

 A deficient or altered circadian rhythm of melatonin secretion (Table 2) was proposed as a mechanism causing excessive daytime sleepiness and depressed mood in PMDD. Some evidence supporting this notion, such as decreases in amplitude, AUC, and mean levels, a phase-advance, and a shorter duration of melatonin secretion in PMDD patients compared to controls were reported [62, 65]. Additionally, when comparing across the menstrual cycle within PMDD patients, onset time was delayed, off-set time was advanced, and duration of secretion was decreased in the LP compared to the FP [65].

Reports of cortisol rhythms in PMDD are inconsistent (Table 2). In one study, PMDD patients showed a tendency for a phase-advance of the cortisol rhythm during the LP compared to the FP [63], whereas in another, it was delayed by ~1 hour in the late-LP compared to the mid-FP in healthy controls but unchanged in PMDD women [68]. Three other studies failed to detect any significant differences between cortisol patterns in healthy controls and PMS/PMDD patients [57, 66, 106]. 

Other hormonal rhythms, such as TSH and PRL, were investigated in PMDD women, though the number of studies is limited (Table 2). The peak time and acrophase of TSH secretion was significantly phase-advanced in patients compared to controls, without any changes in concentration [64]. Throughout the menstrual cycle, amplitude and peak of PRL were higher in PMDD patients compared to controls [63, 64], with a phase-advanced acrophase also detected in these women [64]. In both of these studies, sleep patterns and light-dark exposure were controlled for and stabilized. Nevertheless, TSH and PRL profiles, both of which are affected by the sleep-wake cycle [14], were not obtained under constant conditions (including sleep deprivation); so masking effects cannot be excluded.

### 8.3. Summary and Future Steps

The major findings regarding altered hormone patterns in PMDD include decreased melatonin secretion (AUC and amplitude) (Table 2), which is reminiscent of findings in patients with major depressive disorder (MDD) [107]. Lending further support to the idea that PMDD women experience a phase-advance of circadian rhythms similar to what is observed in MDD [108], these women also experienced a tendency for phase-advanced CBT rhythms as well as significantly advanced melatonin and TSH when compared with controls (Table 2). Since this altered circadian physiology can contribute to an internal desynchrony, resulting in poor sleep quality and mood symptoms, more studies conducted under strict constant routine conditions are necessary. A better understanding of disturbed circadian rhythms in these women may lead to improved chronotherapeutic techniques, which, while similar to those already used in MDD and seasonal affective disorder [109], can be specialized to treat PMDD women.

## 9. Nonpharmaceutical PMDD Therapies Targeting Circadian Rhythms

Treatments of PMDD that target and correct circadian rhythm abnormalities may be an effective alternative to drug-based therapies and may function via a realignment of biological rhythms with the sleep-wake cycle. 

### 9.1. Light Therapy

Since PMDD patients seem to experience a phase-advance of biological rhythms [45, 62, 64], it was hypothesized that light therapy, particularly in the evening, could have therapeutic effects. Indeed, studies have found that light therapy was effective in significantly reducing depressive symptoms in PMDD patients [110–112]. While an initial study by Parry et al. [110] found that bright evening light was more effective than morning light, a follow-up study by the same group [111] achieved similar beneficial effects of symptom alleviation in PMDD patients using bright white light in the morning, bright white light in the evening, and dim red light in the evening (a putative placebo). As the authors point out, a placebo effect cannot be excluded. A study by Lam et al. showed that compared to baseline values, bright white light in the evening was more effective than dim red light in the evening in improving symptoms [112]. This improvement may be achieved via a resynchronization or phase-shift of biological rhythms, since, compared to neutral-dim red light, bright evening light therapy was shown to delay the onset and offset of melatonin [65], increase the midpoint concentration of melatonin [65], delay cortisol acrophase [63], and increase TSH nadir [63] in PMDD patients during the LP.

### 9.2. Sleep Deprivation

Total [113] and partial [114] sleep deprivation (SD) was also shown to be effective in reducing depressive symptoms in PMDD patients, with as many as 80% of patients responding to this treatment [113]. In a series of studies, Parry et al. described the physiological effects of selective SD in PMDD patients [64, 68, 105]. After early-SD (sleep times: 03:00–07:00) during the LP, CBT [105], PRL [64], and TSH [64] acrophases were phase-delayed, PRL amplitude was lowered [64], and TSH amplitude was increased [64]. Late-SD (sleep times: 21:00–01:00) increased CBT amplitude [105] and advanced the acrophase of CBT [105], PRL [64], and cortisol [68], while it delayed the TSH acrophase [64]. Additionally, late-SD also resulted in a decreased PRL mesor [64] and increased TSH mesor [64]. These changes, particularly the phase-delays achieved in CBT and TSH, as well as amplitude changes produced in CBT and PRL, indicate, that like light therapy, SD might achieve its mood elevating effects by targeting and correcting abnormal circadian rhythms. 

A study by Parry et al. demonstrated that, compared to baseline late-LP, both early-SD and late-SD were effective in improving sleep quality in PMDD patients during a night of recovery sleep in the LP. Reference [52] Total sleep time, SE (%), SWS (min), and REM sleep (min and %) were increased, whereas SOL (min), ROL (min), WASO, stage 1 sleep (min and %), and stage 2 sleep (%) were decreased. The authors concluded that these therapeutic effects were accomplished, at least partially, via a correction of altered circadian rhythms which affect the sleep-wake cycle. Responders in this study showed improved mood scores during the LP after early-SD, which were significantly correlated with changes in REM sleep and ROL, indicating REM parameters to be important for the therapeutic effects of SD. The therapeutic effects of SD, however, were only studied during experimental nights and at a single recovery night [52]; therefore the duration of improvement in response to such a treatment is unknown. These results are quite promising, though, so more studies should be carried out along these lines to determine the duration of such positive responses.

### 9.3. Summary and Future Steps

PMDD patients, like those with MDD, have responded favorably to light therapy during their symptomatic LP. Unlike MDD, however, in which morning bright light had the greatest antidepressant effects [109], two studies demonstrated the most mood improvement after evening bright light. 

Studies have demonstrated that 50%–60% of MDD patients respond to SD, with greater effects on mood often observed when SD is restricted to the latter portion of the night [115]. PMDD patients responded with mood improvements after both partial and total SD, and interestingly these treatments often resulted in favorable shifts of circadian physiology. Producing changes in the proper direction to correct for altered rhythms in PMDD, early-SD delayed rhythms of CBT and TSH, and decreased PRL amplitude, while late-SD increased CBT amplitude, delayed TSH and decreased PRL; however it also advanced rhythms of CBT, PRL, and cortisol (not favorable). It should be pointed out that the human circadian system, however, is extremely sensitive to light [116, 117], and since ambient light levels during waking episodes in these experiments were kept at <100 lux, the phase shifting effects of light exposure on these rhythms cannot be excluded. Based on the single study discussed above [52], both early- and late-SD produced improvements in objective sleep parameters in PMDD patients, though future laboratory studies in this direction should address how long these improvements persist beyond a night of recovery sleep. 

Preliminary results from our study investigating the effects of exogenous melatonin taken prior to nocturnal sleep periods during the LP indicate that melatonin may be beneficial in alleviating sleep disruptions in PMDD women [118]. It remains unclear whether melatonin exerts these effects on sleep via a chronobiotic/phase-shifting mechanism, its sedative/soporific properties, a direct action on hypothalamic sleep centers, or some other pathway.

## 10. Conclusions

Evidence from a variety of sources indicates that the menstrual cycle interacts with circadian processes to alter the expression of hormonal rhythms and sleep organization at different menstrual phases. This can lead to sleep alterations during the LP in healthy women or more specific LP-associated pathology like PMDD. 

The most consistently observed menstrual cycle-related changes in the sleep profile of healthy women are a reduction of REM sleep [49, 51–53, 56], with a maintenance of homeostatic sleep mechanisms throughout the cycle [90], and a robust variation of SFA across the menstrual cycle [48, 49, 55], which increases in association with progesterone during the LP. Similarly, women with PMS/PMDD have also shown decreases in REM sleep [52, 56] and increases in SFA [55] during the LP compared to the FP. Sleep complaints during the LP are a symptom of PMDD. PSG-based studies do not consistently demonstrate disrupted objective sleep in PMDD (see Table 1), though some have shown increased stage 2 sleep and decreases in SWS or REM sleep compared to healthy women [45, 46]. 

The circadian variation of CBT is altered by the menstrual cycle in both groups of women. Mean levels are increased (particularly during night time hours) [35, 36] and the circadian amplitude is reduced [35–38] during LP. Some studies have reported further nocturnal increases and phase-advanced rhythms in PMS/PMDD patients compared to healthy women [45, 104]. Generally, circadian hormone rhythms are not significantly altered across the menstrual cycle (see Table 2), though variable results including both increases [58, 59] and decreases [37] in melatonin as well as changes in the timing of hormones [35] have been described. Decreased nocturnal melatonin secretion in PMS/PMDD has also been observed [62, 65]. Finally, nonpharmacological therapies for PMDD symptoms which target the sleep-wake cycle and circadian rhythms, such as phototherapy [110–112] and sleep deprivation [52, 65, 113, 114], are often effective in improving mood and sleep quality in these patients. 

Because of the persistent inconsistencies in the literature, however, it is necessary to conduct more investigations of circadian rhythm changes across the menstrual cycle. These should make efforts to assay sex hormone levels, utilize constant conditions, control for light exposure, and record sleep at numerous points throughout the menstrual and circadian cycles. In light of the present discussion, it is critical that researchers who are interested in including female participants in studies on sleep and circadian rhythms always make efforts to control for and document menstrual cycle phase. If the aim is to observe changes associated with PMDD, participants should also be studied during the symptomatic LP. When including healthy women in general sleep/circadian experiments, it appears better to study them during the mid-FP, in order to minimize interindividual variability in physiological rhythms associated with the LP. Investigations focusing on the interaction between circadian physiology, sex hormones, and the sleep-wake cycle in women across the lifespan will be important to understand the role age-related neuroendocrine changes play in the regulation of sleep and circadian rhythms.

## Figures and Tables

**Table 1:** The variation of sleep across the menstrual cycle.

Authors [Reference]	Year	Sample size	Menstrual phases studied	Significant effect of menstrual phase	Significant effects in PMDD (versus NC)
Parry et al. [45]	1989	*n* = 8 healthy*n* = 8 PMS	early-FP	In healthy and PMS: variation of stage 3 (min) and intermittent awakening across cycle	↑ stage 2 (%) across cycle
			late-FP		
			early-LP		↓ REM (min, %) across cycle
			late-LP		

Lee et al. [46]	1990	*n* = 6 healthy	FP	none	↓ SWS (%) at both phases
		*n* = 7 symptomatic	LP		↓ latency to stage 1 in LP

Ito et al. [47]	1993	*n* = 7 healthy	menses	↓ SWS (min) during early-LP and late-LP	N/A
			late-FP		
			early-LP		
			late-LP		

Ishizuka et al. [48]	1994	*n* = 5 healthy	3 nights/week across full cycle	↑ SFA during late-LP	N/A

Driver et al. [49]	1996	*n* = 9 healthy	menses	↑ NREM (%) during LP	N/A
			early-FP		
			mid-FP	↑ stage 2 (%) during LP	
			late-FP		
			ovulation	↓ REM (% NREM-REM cycle duration) during LP	
			early-LP		
			mid-LP	↑ SFA during LP	
			late-LP		

Chuong et al. [50]	1997	*n* = 6 healthy*n* = 3 PMS	mid-FP	none	none
			ovulation		
			mid-LP		

Baker et al. [51]	1999	*n* = 10 healthy	menses mid-FP mid-LP	↓ REM (%) during mid-LP versus mid-FP ↓ latency to stage 3 during mid-LP versus menses	N/A

Parry et al. [52]	1999	*n* = 18 healthy*n* = 23 PMDD	mid-FPlate-LP	In healthy and PMDD: ↑ ROL, ↓ stage 3 (min and %), and ↓ REM (min) during late-LPIn healthy: ↑ stage 1 (min, %) during late-LP	none

Shibui et al. [37]	2000	*n* = 8 healthy	FP	↑ number of SWS-containing naps during LP	N/A
			LP		

Baker et al. [43]	2001	*n* = 9 healthy	mid-FP	none	N/A
			mid-LP		

Baker et al. [53]	2002	*n* = 13 healthy	mid-FP	↓ REM (%) during mid-LP	N/A
			mid-LP	↑ SWS (%) during mid-LP	

Driver et al. [54]	2005	*n* = 11 healthy	FPLP	↑ stage 2 (%) during LP	N/A

Baker et al. [55]	2007	*n* = 12 healthy*n* = 9 PMS/PMDD	mid-FP late-LP	In healthy and PMS:↑ WASO (min), ↑ microaraousals/hour, and ↑ SFA during late-LP	↑ ROL (min) at both phases

Lamarche et al. [56]	2007	*n* = 8 healthy*n* = 10 PMS	FP late-LP	In healthy and PMS: ↑ stage 2 (%),↓ SWS (%), and ↓ REM (%) during late-LP	none

PMS: premenstrual syndrome; PMDD: premenstrual dysphoric disorder; FP: follicular phase; LP: luteal phase; REM: rapid eye movement sleep; ROL: REM onset latency; NREM: non-REM sleep; SWS: slow wave sleep; SFA: spindle frequency activity; WASO: wake after sleep onset.

**Table 2:** The variation of hormonal rhythms across the menstrual cycle.

Authors [Reference]	Year	Sample size	Frequency of sampling	Hormones sampled	Menstrual phases studied	Significant effect of menstrual phase	Significant effects in PMDD (versus NC)
Steiner et al. [57]	1984	*n* = 2 healthy	2x/hour for 24-hours	plasma cortisol	FP	none	none
		*n* = 2 PMS		plasma PRL	LP		

Webley and Leidenberger [58]	1986	*n* = 10 healthy	1x/4-hour for 24-hours	plasma melatonin	FP	↑ melatonin during LP	N/A
					LP		

Brun et al. [59]	1987	*n* = 9 healthy	1x/night	urinary immunoreactive melatonin	across full cycle	↑ melatonin during LP	N/A

Brzezinski et al. [60]	1988	*n* = 14 healthy	1x/2-hour for 24-hours	Plasma melatonin plasma PRL	early-FP	none	N/A
					ovulation		
					mid-LP		

Berga and Yen [61]	1990	*n* = 10 healthy	1x/hour in daytime 2x/hour in nighttime	Plasma melatonin	early-FP	none	N/A
					late-FP		
					mid-LP		
					late-LP		

Parry et al. [62]	1990	*n* = 8 healthy *n* = 8 PMS	2x/hour for 27-hours	plasma melatonin	early-FP	none	↓ melatonin AUC ↓ melatonin duration melatonin phase-advanced
					late-FP		
					mid-LP		
					late-LP		

Ito et al. [47]	1993	*n* = 4 healthy	1x/hour for 24-hours	plasma melatonin	menses	none	N/A
					late-FP		
					early-LP		
					late-LP		

Parry et al. [63]	1994	*n* = 11 healthy *n* = 21 PMDD	2x/hour for 27-hours	plasma cortisol	mid-FPlate-LP	In healthy: cortisol phase-delayed in late- LP	↑ PRL amplitude at both phases↑ PRL peak at both phases
				plasma PRL plasma TSH			

Cagnacci et al. [35]	1996	*n* = 7 healthy	4x/hour for 24-hours	plasma melatonin	FP	melatonin phase-delay during LP	N/A
					LP		

Parry et al. [64]	1996	*n* = 18 healthy *n* = 23 PMDD	2x/hour for 27-hours	plasma TSH plasma PRL	mid-FPlate-LP	none	↑ PRL peak at both phases TSH phase-advanced at both phases

Parry et al. [65]	1997	*n* = 11 healthy *n* = 21 PMDD	2x/hour for 27-hours	plasma melatonin	mid-FPlate-LP	In PMDD:↓ AUC,↓ amplitude,↓ duration, delayed onset during late-LP	↓ AUC at both phases
							↓ mean levels at both phases

Bloch et al. [66]	1998	*n* = 10 healthy *n* = 10 PMS	1x/day	plasma cortisol	early-FP	none	none
					mid-FP		
					late-FP		
					ovulation		
					early-LP		
					mid-FP		
					late-FP		

Wright and Badia [67]	1999	*n* = 25 healthy	1x/hour for 24-hours	salivary melatonin	FP	none	N/A
					LP		

Shibui et al. [37]	2000	*n* = 8 healthy	1x/hour for 24-hours	Plasma melatonin plasma cortisol plasma TSH	FPLP	↓ melatonin AUC during LP	N/A
						↓ cortisol amplitude during LP	
						↓ TSH amplitude during LP	
						TSH phase-delay during LP	

Parry et al. [68]	2000	*n* = 15 healthy	2x/hour for 27-hours	plasma cortisol	mid-FP	In healthy: cortisol phase-advanced in LP	none
		*n* = 15 PMDD			late-LP		

PMS: premenstrual syndrome; PMDD: premenstrual dysphoric disorder; FP: follicular phase; LP: luteal phase; PRL, prolactin; TSH: thyroid stimulating hormone; AUC: area under the curve.

## Glossary of Abbreviations

PMDD: Premenstrual dysphoric disorder
TSH: Thyroid stimulating hormone
PRL: Prolactin
LP: Luteal phase
SWS: Slow wave sleep
SWA: Slow wave activity
SCN: Suprachiasmatic nucleus
SOL: Sleep onset latency
SE: Sleep efficiency
REM: Rapid eye movement
ROL: Rapid eye movement sleep onset latency
SFA: Spindle frequency activity
vSPZ: Ventral subparaventricular zone
DMH: Dorsomedial nucleus
TMN: Tuberomammillary nucleus
LC: Locus coeruleus
VLPO: Ventrolateral preoptic nucleus
CBT: Core body temperature
FSH: Follicle-stimulating hormone
LH: Luteinizing hormone
FP: Follicular phase
POAH: Preoptic anterior hypothalamus
PSG: Polysomnography
WASO: Wake after sleep onset
NREM: Non-REM
AUC: Area under the curve
PMS: Premenstrual syndrome
MDD: Major depressive disorder
SD: Sleep deprivation
